# On the experience of the sublime in architectural atmospheres: a phenomenological inquiry

**DOI:** 10.3389/fpsyg.2025.1588729

**Published:** 2025-11-11

**Authors:** Benedikte Kudahl

**Affiliations:** Department of Psychology, University of Copenhagen, Copenhagen, Denmark

**Keywords:** sublime aesthetics, phenomenological method, phenomenology of architecture, phenomenological psychology, atmosphere

## Abstract

The concept of the sublime has inspired numerous philosophical inquiries throughout the evolution of aesthetic thought. This article examines the type of experience referenced by philosophers in their discussions surrounding the concept of the sublime. I thoroughly explore the experiential dimensions by analyzing the sublime from a psychological perspective anchored in phenomenology. More specifically, this research concentrates on the manner in which the sublime is perceived within architectural contexts. Through a phenomenological examination of architectural experiences depicted in esteemed literary works, I propose a comprehensive framework detailing the experiential manifestations of the sublime. Finally, I discuss the philosophical, aesthetic, and psychological implications of the sublime experience, as well as its intricate relationship with atmosphere.

## 1 Introduction

The word ‘sublime’ applies in many contexts: it can describe artworks, buildings, landscapes, remarkable achievements, or ideas. Understanding the sublime involves more than just value assessments; it indicates a specific state of mind that arises in reaction to phenomena that are so immense that we struggle to comprehend or express them in language (Shaw, 2017). Currently, the term is rarely used. When we do refer to something as sublime, it is typically in an exaggerated manner, suggesting that something is remarkably pleasant, similar to “excellent” or “wonderful,” but historically, “sublime” has encompassed a much broader meaning than merely indicating something exceptional or highly enjoyable.

Throughout the history of aesthetics, numerous philosophers have shown an analytical interest in the concept of the sublime. Thus, historically, the discourse surrounding its origins has undergone significant transformations (Shaw, 2017; Brady, 2013; Loose, 2011). Religious perspectives suggest that the source of the sublime is God, where the sublime and the divine converge as one. Around the 18th century, idealistic thinkers proposed that the only principle for aesthetically grasping the sublime lies in subjectivity (Gadamer, 2014). On the other hand, romanticists contend that the sublime is the capacity to express the infinite richness of God and nature through the finite creation of the work of art and the act of thinking (Hegel, 1993). One of the most prominent theorists on the sublime, Burke (2008), argues that the sublime is a facet of language, which compels him to reconsider the emphasis previous analyses placed on consciousness. Finally, in a more contemporary context, postmodernists of the twentieth century generally argue that the sublime can only be understood as a manifestation of avant-garde artistic creation.

The diversity of viewpoints is striking, to say the least. I would like to raise the question of what philosophers intend when they engage in discourse regarding the sublime. Is it merely a metaphysical term used to represent a particular system of thought? Or does it refer to a particular type of experience? If so, what is it like to experience the sublime?

One might argue that philosophers do, in fact, address the experience of the sublime in their discussions of the concept. One of the most influential thinkers on the subject of the sublime, Kant, identifies a remarkable experiential aspect of the sublime by stating that our comprehension falls short regarding the sublime. We call sublime that which is “absolutely great,” Kant (1987) explains. Therefore, when aesthetic experiences drift beyond conventional comprehension, and an object’s or event’s significance renders words inadequate, we turn to the idea of the sublime. Kant (1987) further explains that the sublime involves a two-phase subjective reaction: The first phase is marked by a strong sense of fear, which may manifest as an impression of awe, followed by a feeling of satisfying mercy as the power of reason within the subject alleviates the terror experienced in the initial phase. Thus, the sublime stirs both pleasure and pain in equal parts.

While Kant devotes a considerable portion of his of aesthetic judgment to describing how the sublime object appears to the subject, he operates under the premise that the sublime only “exists” as a consequence of transcendental subjectivity. The natural objects that typically “arouse” or “prompt” the sublime—volcanoes, thunderclouds, hurricanes, high waterfalls, and mighty rivers (Kant, 1987, p. 120)—do so only in an illusory manner, constructed through an instance of unconscious deception. The true source of the sublime resides solely within the human subject—nowhere else. Yet we, as humans, assign to nature the sublimity that rightfully belongs to us due to our cognitive ability to confront those natural phenomena that suggest spatial infinity or overwhelming power.

In this paper, I suggest operating under a different premise. We will assume the starting point that the sublime is not merely found “in” subjects nor in specific objects; instead, it envelops us, existing in what Heidegger calls “the aroundness of the environment,” which highlights the spatial nature of Dasein (Heidegger, 1962, p. 134). As Casey (1997) points out, to inhabit an environment or landscape means being surrounded by a series of places rather than just facing a single location. We come to understand the sublime through our engagement, as Casey notes: it is important to realize that it is neither entirely within me nor completely outside of me. To be immersed in a sublime landscape is to exist in a space where I actively participate—interacting with the places around me. Ultimately, we recognize the sublime as situated not solely within the evaluating or emoting subject, nor in the assessed or emotionally impactful objects, but in the complete landscape that is shaped both naturally and culturally, with the subject being an integral part of it.

The goal is thus to explore the experiential aspect by pursuing a phenomenological psychological understanding of the sublime. This article aims to offer a comprehensive insight into (1) the meanings conveyed through the experience of the sublime and (2) the structures of consciousness associated with this experience and their manifestations.

This article begins with the premise that understanding the concept of the sublime is essential to psychology. However, I intentionally do not pose the question, “What is the sublime?” Addressing that question would exceed the article’s focus, as it would require comparing various theories on the sublime put forth by thinkers like Longinus, Burke, and Kant. Philosophers such as these may question the nature of the sublime, asking *what* the sublime is. However, in accordance with phenomenological methodology, I aim to explore how the sublime manifests in experience.

## 2 Method

In this paper, the experience of the sublime is addressed though phenomenological methodology. One of the most interesting lessons from Edmund Husserl’s phenomenological motto “back to things themselves,” is that the primary experience of everyday lifeworld serves as an undeniable source of validity in its own right (Husserl, 1983: § 24). “Experience itself is the givenness of an original datum that excludes any possibility of error by definition: being is what is given to consciousness and conscience fulfills itself in the givenness of being” (De Waelhens, 1953, p. 26, my translation). In this sense, being and experience, givenness and perception, are not dissociated, as experience is ultimately an undeniable kind of presence (Husserl, 1983: § 24).

Since phenomenology has shown that experience itself cannot be doubted, every experience necessarily touches us in a new, unique, and irrefutable way (Gadamer, 2014). The uniqueness and richness of the experiential realm make it essential to use reflection and interpretation as methods to understand the meanings evident in experience.

Relying on phenomenological ideas, I thus believe it is essential to revisit the lived experience of the sublime, as only this foundation enables us to truly comprehend it. When we set aside philosophy, theory, abstraction, and representation, we find the core of our questions and fascination with the sublime: our primary, embodied experience of something sublime. This means that descriptive accounts of sublime experiences, rather than theoretical ones, form the basis of this analysis.

This approach generally differs from historical and most contemporary efforts to capture the essence of the sublime. Exceptions are anthropologist Goble (2016) and philosopher Gallagher (2022), who, respectively, have attempted to convey the experience of the sublime in phenomenological terms. Like them, I assume the sublime can be experienced in various contexts. Goble, for example, describes experiences of visual art, while Gallagher examines experiences of outer space. Generally speaking, the history of the sublime showcases its impact across numerous fields, including art, literature, nature, religion, language, and politics (Shaw, 2017). To delimit the scope of this article, this research focuses on how the sublime manifests within architectural experience.

Architecture provides a unique context for studying the sublime, as the characteristic “greatness” Kant, amongst others, has used to describe the sublime is typically a central feature of prominent architecture. In their reflections on sublime architecture, Nesbitt (1995) and Vidler (1996) inspire our goal in this study of sublime experiences: to cultivate the ability to understand the connections between the mind and the dwelling, the body and the house, the individual and the city.

Another aspect that makes architecture appealing when discussing experiences of the sublime is the apparent distance with which the experience presents itself to the observer, coupled with humanity’s intimate existential engagement with architecture. As the architect and author of the classic study of the experience of architecture, Steen Eiler Rasmussen has pointed out, no other form of art is as cold and abstract; yet, no other form of art is as profoundly connected to day-to-day human experience and our existence from the “cradle to the grave” (Rasmussen, 1962, p. 14). Lived experience is not dissociable from lived space. Architecture has the capacity to develop new forms of lived space on the basis of a very abstract starting point, that is, architectural blueprints and floor plans. These new variants of lived space confront us with a new type of lived experience and a new horizon (Maldiney, 2022).

Keeping this in mind, I assume that architecture, while a cold and abstract concept, becomes warm and vital as it significantly influences the subjects experiencing its forms, cavities, atmospheres, etc.

To capture the experience of the architecturally sublime, I will address its bodily manifestation. My research into the architectural sublime thus aligns with the position of Pallasmaa (2012) in supposing that scholars often underestimate the roles of touch, smell, taste, movement, and hearing in architectural experiences. Unlike contemporary architecture, which, like much philosophy, has strong “ocular-centric tendencies,” Pallasmaa advocates an all-senses approach. “Sight is the sense of the solitary observer” (Pallasmaa, 2012, p. 54), he says, while other senses put us in direct interaction with a given space. For instance, he states that space is both understood and appreciated through its echo as much as through its visual form. He emphasizes how the often-unnoticed background presence of auditory experience adds a specific emotional depth to the architectural experience because “the sound reverberating from surrounding walls puts us in direct interaction with space” (Pallasmaa, 2012, p. 55).

If we accept that hearing gives architectural experience a sense of connection to space, describing architectural experience solely in terms of sight would be problematic: without the auditory element, the description would lack rigor and depth. The same applies to the senses of touch, smell, taste, and movement. While I assume that experiences of architecture involve the entire body, certain forms of aesthetic experience (in this case, those described as sublime) may highlight specific modalities while leaving others buried in the pre-reflective and, thus, unarticulated realms.

I aim to explore the engaged modalities (along with other structures of consciousness that facilitate meaning creation) by analyzing descriptions of experiences of the sublime in architectural contexts. Thus, I base this phenomenological investigation of the sublime on an analysis of literary works by notable writers and poets that describe the experience of the sublime within architectural spaces. I will use these descriptions to capture the aesthetic uniqueness of the sublime as it manifests in architectural contexts.

Applying Husserl’s phenomenological method within a psychological research context, I take inspiration from Giorgi’s (2009) descriptive phenomenological psychological method. In line with Giorgi, I seek to articulate the psychological meanings of lived experiences by analyzing in-depth descriptions of how the phenomenon under investigation presents itself to the experiencing subject. Building on Husserl’s philosophy, Giorgi maintains that the objective is to understand how phenomena present themselves to consciousness, highlighting how description facilitates the clarification of these processes.

Thus, I strive to adhere to Husserl’s method of phenomenological reflection and apply this rigorous approach to descriptions of sublime experiences present in literary works by esteemed authors. Specifically, I have chosen extracts from four pieces for analysis: *Mrs. Dalloway* by Virginia Woolf, *The Heights of Macchu Picchu* by Pablo Neruda and Henry Miller’s *Colossus of Maroussi* and *Tropic of Capricorn, respectively.* These literary works are mainly selected for their rich descriptions of powerful architectural experiences. They are all written in a style that aims to convey subjective experience to the reader, and they are all set in notable architectural contexts. Woolf’s book is filled with celebrated descriptions of London’s architectural metropolis, assembling Miller’s *Tropic of Capricorn*, which depicts the city of New York. Both Neruda’s poetic tribute to Machu Picchu and Miller’s *Colossus of Maroussi* are recognized testimonies to historical architecture. While only some of the selected sections directly use the term “sublime” to describe architectural experiences, these particular sections are highlighted because they conform with Kant’s idea that sublime experiences are aesthetic experiences in which the subject’s cognitive comprehension falls short and which stir both pleasure and pain in equal parts.

By conducting a phenomenological analysis of selected descriptions of architectural experiences in these literary works, I intend to uncover a general experiential structure present in all of them. The aim of this phenomenological analysis of these pieces is thus to capture how experiences of the sublime generally appear in the context of architecture. I do so by employing Husserl’s principle of phenomenological reduction. This process requires transitioning from the natural attitude we adopt in our everyday lives to a phenomenological attitude, which permits a more rigorous viewpoint. This shift arises from the act of “bracketing past knowledge or unrepresented assumptions regarding the object at hand” (Giorgi, 2009, p. 91) that the reduction entails. It is crucial to understand that bracketing our prior knowledge about the sublime does not mean forgetting; instead, bracketing involves intentionally refraining from engaging with that knowledge (Husserl, 1983).

Furthermore, I applied Husserl’s (1983) principle of imaginative variation to each description of experience drawn from the literary works. As Giorgi highlights, “particular situations encompass various contingencies, only some of which are essential for genuinely grasping what is typical in these contexts” (Giorgi, 2009, p. 84). This underscores the necessity of altering these specific situations to uncover a stable phenomenological structure within the descriptions. Therefore, the imaginative variation of the particular content of the descriptions enables the general structure to extend beyond the specific, individual situations, allowing the phenomenon itself to begin to emerge. By applying this principle in this analysis of literary descriptions, I sought to establish a general descriptive structure of the sublime architectural experience, ultimately revealing the essential features of the sublime phenomenon.

## 3 Results

The analysis reveals how the architecturally sublime is configured in experience. I present this configuration by describing what appears to be four different main components of the phenomenon of the sublime as it appears in the context of architecture. These four interacting components are not abstract pre-established analytical categories. Instead, in line with Giorgi’s (2009) method, they are experiential constituents that emerge from analyzing the selected literary descriptions of how the sublime appears in the context of architecture. For further research, a preliminary hypothesis is that the essential features of this structure of experience would be recognizable even in descriptions of sublime experiences that occur in contexts unrelated to architecture.

As for all experience, the experience of the sublime cannot be reduced to a list of individual components; rather, it is a dynamic configuration of forces that exist in relation to one another (Husserl, 1983). Collectively, these interactions shape the experience of sublime architecture. To provide an overview, however, the interactions can be enumerated as follows:

1. Architectural entrance: Elevation
2. Mortal atmospheres of elevated places
3. Images of mortal suffering
4. Discovery: Understanding through suffering

Observe how each “step” is linguistically linked to the previous one. Instead of being a coincidence, I connect each part through the repetition of words (elevate, mortal, suffering) to emphasize the interrelated nature of the various aspects of the phenomenon.

One purpose of this analysis has been to understand the meaning with which the architecturally sublime appears through these four aspects and the dynamic relationships between them. Another (yet truly interlinked) purpose has been to pinpoint the experiential structures of consciousness through which these meanings appear. My analysis of these reveals an order of *how* subjectivity is involved in the creation of the meaning of the sublime.

1. Upward movement and affectivity
2. Multiple co-occurring forms of sensory perception and affectivity
3. Visual imagery and affectivity
4. Reflection

The structure of these experiential aspects should be considered alongside the previously mentioned structure of meaning. In this context, each element of meaning emerges from its corresponding feature of consciousness. Similar to the first structure, the ladder cannot be simplified into a collection of individual elements; it is presented this way to provide an overview.

### 3.1 Architectural entrance: elevation

A historical definition of elevation refers to the act of raising or building something up “to rear or raise by building,” (‘elevation, n.’ *OED Online*, Oxford English Dictionary, 2025a). One could argue that “to elevate” is the most straightforward illustration of humanity’s defiance of nature: the difference between being on the ground and being uplifted, even if it is to a minor degree. Hence, the phenomenon of elevation appears closely tied to construction, building, and architecture.

In my reading of the literary pieces mentioned, I find that the key for the experiencing subject to access the architecturally sublime is the feeling of elevation provided by architectural objects. This feeling of elevation seems to grant the subject entry into another experiential realm. It is not that the feeling of elevation is inherently sublime; instead, entering this other experiential realm is the initial condition for the architectural sublime to eventually manifest. The act of entering is, however, a particularly critical moment, as without it, the dynamic of the subsequent constituents would not be able to unfold.

Not all architecture conveys a sense of elevation; however, my analysis reveals that various forms of architectural construction can potentially create a feeling of elevation. My analysis shows that the feeling of elevation can emerge from interactions with architecture ranging from the simplest and most mundane forms of elevated architectural construction (a second-floor window, a ladder, high stoops with banisters) to the more advanced and even architecturally recognized (a temple on a mountain hill, skyscrapers, the elevated train going across Manhattan Bridge). In Neruda’s (2014) poem, the elevated object is the 15^th^-century city of Machu Picchu. Neruda visited Peru’s “lost city of the Incas” when it was rediscovered. The city’s wonders and mysteries are first presented to the narrator upon a steep mountain climb:

Then up the ladder of the earth I have risen,

through the awful tangles of lost forests,

up to you, my friend, Macchu Picchu.

High city of laddered stones,

at last, final dwelling for those whom earthliness

did not hide in its sleeping garments.

Neruda’s poem exemplifies the various and complex meanings of the elevation phenomenon. First, the “high city” of Machu Picchu is elevated in the perhaps most straightforward meaning of elevation: it is geographically raised or lifted to a particular height above a given level (above sea level or the ground around the mountain tops). In the poem, the narrator provides additional meaning to the sense of a place “raised” by describing the revelation of the raised place through the upward movement leading the discoverer’s body to the high city. In other words, it is not just the city that is raised, but it is also the “I” that has “risen,” that is, which has *moved upward*.

The poem thus exemplifies how the bodily experience of upward movement contributes to the experience of the architectural sublime as a feeling of elevation. In this context, I argue that the body must be understood as a lived body (Merleau-Ponty, 2012), indicating that it is a bodily subject experiencing the sensation of being lifted to a higher place. This means that the feeling of elevation is not just a physiological fact but also an emotional experience. In an emotional sense, “elevation” signifies being “lightened” or “lessened” in some way (‘elevation, n.’ OED Online, Oxford English Dictionary, 2025a). Neruda’s poem highlights this aspect of feeling elevated through the imagery of the “awful tangles of lost forests” that the “I” is now leaving behind as he ascends to the place of the ancient “friend.”

Some of the most recent phenomenological research tends to confirm this preliminary intuition about the experience of elevation. In his phenomenological analysis of vertical experience, Anthony Steinbock (2007) has highlighted that various phenomena exist that cannot be approached through the lens of the noema-noesis scheme proposed by Husserl (1983). There are certain phenomena whose givenness will result in a “subjective provocation” (Steinbock, 2007). The givenness of these phenomena is beyond reach, beyond control, and as a result, these phenomena are not directly graspable. Steinbock (2007, p. 13) calls such phenomena “vertical.” Experiences such as hope, vocation, epiphanies, revelations and the elevation of the sublime aesthetic experience all have the phenomenological structure of verticality.

Although rarely used in modern language, “elevation” also refers to being emotionally uplifted, as when someone “is elevated in their heart” (‘elevation, n.’ *OED Online*, Oxford English Dictionary, 2025a). While this specific form of elevation does not manifest directly, the poem suggests a lifting of the heart, evoking a sense of being spiritually or even morally elevated through the ascent to the “high city.” There is something almost “woken” about the “high city,” which, in contrast to the “earthliness” of contemporary societies, does not clothe their dead in “sleeping garments.” In fact, the spiritually “elevated” place of Machu Picchu is, in Neruda’s description, characterized precisely by the presence of those who have passed away there.

This brings us closer to understanding how the feeling of elevation connects to the second crucial aspect of the “architecturally sublime”: its atmosphere. Aside from the meanings of “elevation” already mentioned, another lesser-known historical meaning of elevation reveals how the phenomenon of elevation is etymologically entangled with sublime atmospheres. Elevation refers to the action of heat: “to raise in the form of vapor; to evaporate or sublime” (‘elevation, n.’ *OED Online*, Oxford English Dictionary, 2025a). Sublimation, in a chemical sense, means the action or process of converting a solid substance directly into vapor through heating, without liquefaction or decomposition (‘sublimation, n.’ *OED Online*, Oxford English Dictionary, 2025b). When applied to the phenomenon of the architecturally sublime, this suggests that the initial experience of elevation through architecture transforms the solid substance of the architectural construction into vapor, essentially diffusing it into the air. Figuratively, such a substance (e.g., mist or fog) implies that atmospheric phenomena characterize the experience. Next, we will explore how the analyzed literary works depict the specific atmosphere of the sublime.^1^

### 3.2 Mortal atmospheres of elevated places

I said above that the initial “architectural elevation” opens and thus gives the subject access to another experiential realm. This realm is characterized by what I will refer to as “mortal atmospheres.” Before discussing this concept, I will review some of the key phenomenological literature on the notion of atmosphere.

As pointed out by Minkowski (1999) and Schmitz (2024), atmosphere can generally be understood as the spatial, emotional, cultural, and situational stage where our lived-presence unfolds. The origins of the specifically *phenomenological* discussion of atmospheres however, can be traced back to Jaspers (1948) phenomenological psychopathology. Jaspers (1948) understands “atmosphere” as that quality to attract the intentional attention towards a particular aspect or element of a certain situation. For example, if we enter a room where an interview is being recorded, our intentional act will be constituted thanks to the object that almost spontaneously has the atmospheric power to draw our attention: the interview.

In spite of the fact that Jaspers’s (1948) concept of atmosphere is very seductive –it can even hide a possible answer to the unresolved problem of the choosing of the object in Husserl’s (1983) theory of constitution-, Jaspers’s understanding of atmosphere seems insufficient in order to comprehend the affective dimension of atmospheres, as well as the affective stimmung that arises in different situations with different atmospheres. This is why Fuchs (2013, p. 617) has pointed out that: “In comparison to existential feelings or moods, affective atmospheres are often felt more distinctly, since they are not experienced as something one carries with oneself, but rather encountered as an enveloping aura, radiating or emanating from the space or environment that one enters.”

In this sense, the phenomenological discussion of the concept of atmosphere highlights the fact that the distinction between subject and object is much richer than classic theory of knowledge suggests and, as shown by Fuchs (2013, p. 616) it:

may be regarded as holistic affective qualities of experienced spatial and interpersonal situations, integrating their expressive features into a unitary dynamic Gestalt: for example, feeling the hilarity of a party, the sadness of a funeral march, the icy climate of a conference, the awe-inspiring aura of an old cathedral, or the uncanniness of a somber wood at night. Such atmospheric effects are evoked by physiognomic or expressive qualities of objects as well as by intermodal features of perception such as rhythm, intensity, dynamics, etc. Like all affective phenomena, atmospheres are experienced through a resonance of the body (an icy atmosphere feels chilly, an uncanny situation makes one shiver or “one’s hair stand on end,” a tense interpersonal climate is felt as oppressive or suffocating, etc.).

Fuchs outlines the affectivity of atmosphere. The question, in the context of this paper, is how the affectivity of atmosphere relates to the aesthetics of atmosphere. We can already find a preliminary answer to Gaston Bachlard’s work. As shown by Bachelard (2014), the study of atmosphere is a key tool in order to investigate human affectivity: the project of a “poetics of space” reveals how space is lived and thus how it is qualitatively experienced beyond the objectivistic boundaries set by geometry and functionality. Space as such can only be fully understood thanks to a prior inquiry of attuned space, that is, thanks to the experience of lived, affective, felt, colored space. And the only way to fundamentally describe the phenomenon of attuned space it is, not by the cold method of scientific measurement, but through a poetics of space and its atmospheric resonances.

More decisively, in the context of aesthetics, as Gernot Böhme points out, the term atmosphere frequently appears in aesthetic discourse (e.g., referring to the powerful atmosphere of a work of art, the atmospheric effect of a play, the atmospheric impression of a particular urban design, etc.), yet while the term is mentioned, the description of that which it signifies is often left out, “as though *atmosphere* were tasked with designating something indefinite” (Böhme, 2017, p. 13). This observation reflects what has been described as an ineffable quality as a constitutive part of atmospheric phenomena (Dufrenne, 1973). As Anderson (2009) puts it, atmospheres both exist and do not exist. On the one hand, the subjects that experience atmospheres need to ‘fulfill’ them for them to appear: It is as if they exist as an extension of the spectator’s affective life. However, on the other hand, atmospheres radiate from the collection of components that comprise the aesthetic item: They are a part of the aesthetic thing (Anderson, 2009).

This tension in the term has also been emphasized by Binswanger, one of the pioneering philosophers of the concept of atmosphere. Binswanger articulates atmosphere as the affective space (“Gestimmtheit”) wherein the individual and the world establish a fundamental unity, thereby enabling the individual’s world to be recognized as his own (Binswanger, 1933). Thus, Binswanger thematizes the affectivity inherent in the relationship within which subjects perpetually exist: the affectively open world-I relation.

In this sense, atmosphere refers to affect felt “in between” the lived body of the experiencing subject and the experienced character of the encountered space.^2^ When examining atmospheres, we refer to their character, says Böhme. “The character of an atmosphere is the way it communicates a feeling to us as participating subjects.” (Böhme, 2013b, p. 2). For example, a solemn atmosphere generally induces a serious mood, whereas a cold atmosphere evokes a sense of chill.

Finally, in my effort to summarize some crucial aspects of atmospheric phenomena, I want to emphasize another key point: atmospheres are experienced in multisensory ways. Thus, vision alone, it can be argued, is insufficient to understand how bodies sense atmospheres.

As previously mentioned, within the analyzed literary depictions of sublime architectural experiences, the initial feeling of elevation grants the individual entry to an alternative experiential realm distinguished by a particular atmosphere. Now, to continue the analysis of the sublime architectural experience, I will analyze the atmospheric quality of the experience by addressing the following aspects: (a) how this atmosphere is identified as precisely that, an atmosphere, (b) the specific character of the atmosphere of sublime architecture, and (c) how this atmosphere appears to the experiencing subject (in terms of the structures of consciousness facilitating its meaning creation). Since these elements are closely interconnected, the following sections will address all three collectively instead of treating them as separate entities.

To explore the atmospheric aspect of the architecturally sublime phenomenon, I will first examine a passage from *The Colossus of Maroussi,* where Miller’s narrator visits the famous ancient Greek archaeological site of Delphi, which is set on the elevated crest of a mountain. Upon arriving at the site, the narrator is struck by a uniquely enchanting atmosphere, prompting him to contemplate the lives of those who once inhabited the ancient city. This historical location leads him to have an experience that transcends the tangible walls of the architecture of Delphi, as he is enveloped by a feeling of timelessness. He describes the atmospheric impression that the site conveys:

Set just below the crest of the mountain one has the impression that when the course was finished the charioteers must have driven their steeds over the ridge and into the blue. The atmosphere is superhuman, intoxicating to the point of madness. Everything that is extraordinary and miraculous about Delphi gathers here in the memory of the games which were held in the clouds. As I turned to go I saw a shepherd leading his flock over the ridge; his figure was so sharply delineated against the sky that he seemed to be bathed in a violet aura; the sheep moved slowly over the smooth spine in a golden fuzz, as though somnolently emerging from the dead pages of a forgotten idyll … one is ever filled with the sense of eternality which is expressed in the here and now (Miller, 1950, p. 198).

Delphi, a place of elevation (at the crest of the mountain), is depicted by Miller as possessing a “superhuman” and “intoxicating” atmosphere, and he elaborates on the vapor of the site, referring to Delphi’s miraculous nature as being “held in the clouds.” Thus, the elevated impression of the architecture is represented through more than just the solid matter of stone; the place’s extraordinary nature manifests through the site’s vaporous air.

The final part of the description emphasizes the unique quality of the atmosphere of sublime experiences, which I have identified and labeled as “mortal atmospheres.” As the sheep are guided over the ridge of the mountain, the air surrounding them is filled with a “golden fuzz,” which is “emerging from the dead pages of a forgotten idyll.” The atmosphere of the elevated site at Delphi is not only miraculous but also imbued with a palpable sense of the vanishing of a once-harmonious past. Although the site of Delphi still exists and is accessible for Miller’s narrator to visit, the living beings of the present seem imbued with an aura of a lost past. Whatever occurred historically at Delphi is now relegated to history. In other words, the place evokes a sense of mortality and, therefore, the fragility of all beings, events, and ultimately moments in time: the mortality of everything that is temporal. The past manifests at Delphi not as a replay of former events but as a sense of historical significance permeating the atmosphere of the site.

I seek to elucidate the subjective perception of this “mortal atmosphere”; specifically, how does the subject who experiences this atmosphere come to acknowledge that this is indeed their experience?

For one thing, this atmosphere seems to diminish the distance perceived between the subject himself and the elevated place where he now finds himself. In another passage from *The Colossus of Maroussi* Miller describes the diminished distance between the narrator and the architecture of the Greek island of Hydra:

Hydra is a rock which rises out of the sea like a huge loaf of petrified bread … Having touched this rock I lost all sense of earthly direction. What happened to me from this point on is in the nature of progression, not direction. There was no longer any goal beyond — I became one with the path. Each station henceforth marked a progression into a new spiritual latitude and longitude (Miller, 1950, p. 60).

The experience of becoming “one with the path” and losing a sense of direction illustrates a bodily subject whose boundaries are blurred. However, the body is not numbed; rather, it is exactly through the activity of the bodily senses that the atmosphere is registered and felt.

This appears to occur through various sensory modalities. In Miller’s description of Delphi, the narrator primarily conveys visual aspects of color (blue or gold) and tactile qualities (smooth or sharp), as well as elements of movement (slowly or bathed). That is, from the analysis of the literary pieces, it seems that the “mortal atmosphere” of the architecturally sublime can be perceived through various sensory modalities (visual, tactile, movement, auditory, olfactory). For example, another excerpt from *The Colossus of Maroussi* illustrates how this atmosphere is perceived as a combination of auditory and visual impressions. Exploring the historical Greek society of Kalami, the narrator moves up along the atmospheric path leading him toward an elevated ancient mountain village. Faced with the hazy landscape, the narrator grapples with the sorrows of the past (not just his own but humanity’s sorrows), evoking a profound sense of loneliness that simultaneously offers a new perspective on humanity’s historical transformations. In this passage, Miller’s narrator illustrates the ambiance of the elevated setting that ultimately inspires this insight:

Finally we came to the path that led up the mountain side, a sort of gully rather than path, which taxed even the mountain ponies on which we had loaded our things. As we climbed a weird melody greeted us from above. Like the heavy mist sweeping up from the sea, it enveloped us in its nostalgic folds and then as suddenly died away. When we had risen another few hundred feet we came upon a clearing in the midst of which was a huge vat filled with a poisonous liquid, an insecticide for the olive trees, which the young women were stirring as they sang. It was a song of death which blended singularly with the mist-laden landscape (Miller, 1950, p. 22).

The auditory impression of the haunting melody sung by local women, combined with the visual allure of the misty landscape, establishes a unique atmosphere. These sensory elements converge to form “nostalgic folds,” as the mist-shrouded scenery and the women’s “song of death” evoke a long-forgotten past, reminding the subject of the delicate nature of mortal existence.

Aside from the multiple sensory modalities involved in the perception of the atmosphere and its specific character of “mortality,” a final crucial aspect of this atmosphere is that the subject—throughout all the literary descriptions—enters it alone. Even if the narrator has company upon arriving at the atmospheric site, the impression of “mortality” in the air remains unshared.^3^ Upon entering, the subject thus appears to encounter a sense of emptiness, loneliness, or solitude. In *Tropic of Capricorn*, Miller paints a bleak picture of the harshness of American society in the 1920s, where the narrator’s solitude is closely linked to the city of New York. In this solitude, the narrator immerses himself in New York’s atmosphere and faces the reality of suffering amid the city’s coldness, emptiness, and despair. In a moment of insight, he understands that there is no escape from this state, no ultimate redemption.

Again the night, the incalculably barren, cold, mechanical night of New York in which there is no peace, no refuge, no intimacy. The immense, frozen solitude of the million-footed mob, the cold, waste fire of the electrical display (…) To walk meaningless and unfecundated through the bright glitter of the calcimined streets, to think aloud in full solitude on the edge of madness, to be of a city, a great city, to be of the last moment of time in the greatest city in the world and feel no part of it, is to become oneself a city, a world of dead stone, of waste light, of unintelligible motion, of imponderables and incalculables, of the secret perfection of all that is minus (Miller, 2015 p. 99).

This discovery leaves the narrator feeling not crushed but clear and eventually humbly empowered as it becomes evident that this harsh yet vital insight could only have emerged from a deep sense of solitude. In her stream-of-consciousness London portrait, *Mrs. Dalloway*, Woolf similarly conveys a sense of solitude at the core of sublime experience as the main character, Clarissa Dalloway, navigates the social gathering she is hosting for part of the London elite. She is the charming and cherished host of a splendid party filled with notable guests. However, she cannot help but feel a sense of loneliness from the two vacant chairs, once occupied by distinguished attendees who had been conversing there just moments ago. In this solitary moment, the character Clarissa Dalloway immerses herself in the mortal atmosphere of the sublime experience that will eventually hit her.

She went on, into the little room where the Prime Minister had gone with Lady Bruton. Perhaps there was somebody there. But there was nobody. The chairs still kept the impress of the prime minister and lady Bruton, she turned deferentially he sitting four-square, authoritatively. They had been talking about India. There was nobody. The party’s splendor fell to the floor, so strange it was to come in alone in her finery (Woolf, 1922, p. 279).

The impression of “nobody” repeats, leaving the reader wondering if Mrs. Dalloway has any real option but to enter that unsettling, atmospheric realm, even though her experiences there promise discomfort. The character is approaching the understanding that all existence inevitably entails some suffering and that death remains the only true escape. Within this solitary mortal atmosphere, both Woolf’s and Miller’s characters—potentially any individual encountering the architecturally sublime—will confront what I refer to as “images of suffering.”

### 3.3 Images of mortal suffering

Existentialists such as Kierkegaard, Dostoevsky, Nietzsche, Heidegger, and Sartre all consider suffering essential to existence, exploring themes such as anxiety, dread, tragedy, anguish, loneliness, and despair. We might say that, as mortal beings, every human undergoes suffering, irrespective of its origin, whether from the act of living or the inevitability of dying. Upon analyzing the literary descriptions of the architecturally sublime, it appears that the “mortal atmosphere” of the “elevated place” (section 3.1–3.2) activates the subject’s imaginative capacity and consequently evokes images of such suffering.

Neruda’s poem refers to the people who lived and died in Machu Picchu’s “high slaughterhouses”, illustrating his imaginative yet empathetic bond with them. The narrator contemplates the origins of Machu Picchu, recognizing the tears and blood of the enslaved individuals who suffered for its creation.

Macchu Picchu, did you place

stone on stone,

and, at the base, rags?

Coal on top of coal, and tears at the bottom?

Fire atop gold, and, trembling in it, the giant red

raindrop of blood?

Return to me the slave you buried!

Shake the suffering people's hard bread

from these lands, show me the servants'

clothes and their windows.

Tell me how they slept when they were alive.

Tell me if, when they slept,

they snored with mouths ajar like black holes

fixed by fatigue on their walls.

Amidst the walls of Machu Picchu, the narrator envisions the lives of its past inhabitants: “Show me!” he demands, longing to glimpse the everyday existence of the sleeping, breathing, snoring servants. His tone is a blend of shock and empathy: the lines express both the sorrow and pain of “witnessing” these fates within his mind while also showcasing his deep concern and connection with those who endured suffering at this remarkable architectural marvel.

The “mortal atmosphere” (3.2) leads to this third moment of the experience by evoking images of places such as slaughterhouses, torture chambers, and other symbolic sites of suffering. Through these images, the subject suddenly becomes almost alarmingly aware of the suffering that may have taken place at this architectural site. By means of this imaginative act, the subject is able to establish a feeling of connection, closeness, or sympathy with these suffering individuals. In *The Colossus of Maroussi,* Miller’s narrator describes his impression of the brutality of ancient Greek society as emanating from the cliffs near a mountain village. As he walks on the elevated plateau, he envisions wading through a sea of blood, perhaps from those slain and left to rot on this very path. However, from this place of carnage and through the image of suffering, a fresh vision of clarity, sympathy, and connection eventually arises in the narrator.

Then the pass, which I shall always think of as the carrefour of meaningless butcheries. Here the most frightful, vengeful massacres must have been perpetrated again and again throughout the endless bloody past of man. It is a trap devised by Nature herself for man’s undoing. Greece is full of such death-traps (Miller, 1950, p. 23).

In both descriptions–of impressions formed within the context of Peruvian and Greek historical architecture, respectively–the suffering is depicted through imagining a distant past. The narrator envisions the violence of this past, empathizes with those who endured it, and seems to ask under his breath, “why?” as if desperately seeking answers. Yet, he concludes that the suffering was entirely pointless. The envisioning, again, is described as an act of visual imagination. This visual-imaginative aspect is perhaps most clearly exemplified in Woolf’s description of Clarissa Dalloway’s day. At the party she hosts for London’s elite, a guest, Lady Bradshaw, informs Clarissa that a young man from their social circle had just jumped out of a window. Lady Bradshaw’s husband learned the news just before Clarissa’s party, and now they are discussing the suicide in the midst of her celebration. Suddenly, Clarissa, overcome by a profound sense of solitude, envisions the young man falling, with the ground rushing up toward him:

What business has the Bradshaws to talk of death at her party? A young man had killed himself. And they talked of it at her party–the Bradshaws, talked of death. He had killed himself—but how? Always her body went through it first, when she was told, suddenly, of an accident; her dress flamed, her body burnt. He had thrown himself from a window. Up has flashed the ground; through him, blundering, bruising, went the rusty spikes. There he lay with a thud, thud, thud in his brain, and then a suffocation of blackness. So she saw it (Woolf, 1922, p. 280).

Like the narrators mentioned above, Woolf’s subject, Clarissa Dalloway, senses the deceased through visual imagery: “so she saw it.” This extract stresses what we have already seen in the texts highlighted above: the subject to whom the phenomenon of the sublime eventually appears is, at this point in the experience, somewhat frightened yet somehow forced to accept the imaginatively witnessed expression of suffering.

### 3.4 Discovery: understanding through suffering

At this point, in the elevated, atmospheric imaginary witnessing of suffering (3.1–3.3) the subject appears to have transcended the ordinary distinction between life and death as good and bad, respectively. Woolf’s story demonstrates this aspect: in the moments following the imagining of suffering, the young man falling from the window, his body being penetrated by “rusty spikes,” Mrs. Clarissa Dalloway contemplates the life and death of the young man who jumped from the window. She conveys to the reader how she perceives life and death as equal states of overwhelm, incapacity, and solitude.

Death was defiance. Death was an attempt to communicate; people feeling the impossibility of reaching the center, which, mystically, evaded them; closeness drew apart; rapture faded, and one was alone. There was an embrace in death … Then (she had felt it only this morning) there was the terror; the overwhelming incapacity, one’s parents giving it into one’s hands, this life, to be lived to the end, to be walked with serenely (Woolf, 1922 p. 280).

Through this reflection, she arrives at one thought: gratitude, not just for life, but for either part of existence. She no longer perceives death as inherently gruesome.

… the words came to her, Fear no more the heat of the sun. She must go back to them. But what an extraordinary night! She felt somehow very like him—the young man who had killed himself. She felt glad that he had done it; thrown it away. The clock was striking. The leaden circles dissolved in the air (Woolf, 1922 p. 293).

Anxiety takes on a new meaning: overcoming the inherently human fears of nature (i.e., the sun) and death (i.e., the young man who has thrown his life away). Woolf’s character has now experienced something that propels her beyond ordinary human anxiety. More broadly speaking, at this point in the manifestation of the sublime, the subject experiences an existential transformation. Having entered the elevated place (3.1), having confronted the phenomenon of mortality through atmosphere (3.2), and having imaginatively witnessed human suffering (3.3), the subject has transcended the ordinary human condition of mortal anxiety. By candidly facing the depths and suffering of existence, the subject has ascended to a state beyond the existential realities of mortality, nature, time, and space, thus overcoming ordinary human fear. The concept of “rising above,” evoked by the initial impression of an “elevated” movement, now acquires a more profound significance. Perhaps it is in this moment of realization that the subject comprehends the essence of this elevated architectural experience.

From this point, the subject intuitively grasps something new. The most profound and gloomiest atmosphere surrounds the subject, yet shaken by wonder and fascination, the subject is now on the verge of making a new discovery about human existence. In *Tropic of Capricorn*, Miller’s narrator, having observed the harsh realities faced by workers in the unforgiving New York of the 1920s, rises above ordinary existence and uncovers the profound nature of human isolation while ascending the stairs from the subway to the streets of this brutal city.

The world I knew is no more, it is dead, finished, cleaned up. And everything that I was is cleaned up with it. I am a carcass getting an injection of new life. I am bright and glittery, rabid with new discoveries, but in the centre it is still leaden, still slag. I begin to weep—right there on the L stairs. I sob aloud, like a child. Now it dawns on me with full clarity: you are alone in the world! You are alone … alone … alone. It is bitter to be alone … bitter, bitter, bitter, bitter. There is no end to it, it is unfathomable, and it is the lot of every man on earth, but especially mine … especially mine. Again the metamorphosis (Miller, 2015, p. 191).

The world he once inhabited has ended, and this transformation includes his identity. What does this new reality entail, and who is he now? A fresh life has been “injected” into him, and this altered version is acutely conscious of the loneliness inherent in existence. This “new life” appears to acknowledge the undeniable constraints of human existence. The description conveys a profound sense of rebirth, a sentiment that resonates throughout the additional descriptions. In *The Colossus of Maroussi*, we find an example of a variation of such discovery of a “new world” made through the architecturally sublime ancient structures in the Greek city of Eleusis:

One must throw off two thousand years of ignorance and superstition, of morbid, sickly subterranean living and lying. One must come to Eleusis stripped of the barnacles which have accumulated from centuries of lying in stagnant waters. At Eleusis one realizes, if never before, that there is no salvation in becoming adapted to a world which is crazy. At Eleusis one becomes adapted to the cosmos. Outwardly Eleusis may seem broken, disintegrated with the crumbled past; actually Eleusis is still intact and it is we who are broken, dispersed, crumbling to dust. Eleusis lives, lives eternally in the midst of a dying world (Miller, 1950, p. 49).

Once again, a world is fading as a new one emerges. To be clear, it appears that the world being “born” is not an ancient world reborn but a fresh vision in which the current world integrates the experiences, wisdom, and pain of past lives. Neruda conveys this message as he describes how the narrator feels the resonance of the suffering that happened in the structures of Machu Picchu. In this passage, he talks of these people as a “brother” of the past. Through connecting with the brother of the past and feeling his pain—not in the flesh, but in spirit—new life is created. A sense, again, of rebirth:

Rise up in birth with me, my brother.

Give me your hand out of the deep

zone of your wide-spread sorrow.

You will not return from the bedrock depths.

You will not return from subterranean time.

It will not return, your hardened voice.

They will not return, your pierced eyes.

Look at me from the depths of the earth, you,

the farm worker, the weaver, the quiet shepherd,

the tamer of guardian guanacos,

the mason on his defied scaffolding,

the water carrier bearing Andean tears,

the jeweler with crushed fingers,

the farmer trembling among his seeds,

you, the potter poured in your clay,

all ye, bring to the cup

of this new life

your ancient buried sorrows.

It is as though the history of sorrow is being integrated into the subject’s current existence. The human of the past does not “return,” as Neruda says. His eyes and voice do not return. The integration of the past occurs not at the level of the flesh but on an atmospheric level. Through experiencing what I describe as “mortal atmospheres, “the subject integrates and sublimates historical pain and sorrow, discovering this “new life” and, consequently, a new vision of things.

## 4 Discussion

This article aims to explore the architecturally sublime through experiential means. Specifically, I seek to introduce a fresh perspective on understanding the sublime - one that does not simplify its description to a transcendental ability for fearless contemplation within the observer (Kant, 1987: §28), a linguistic characteristic (Burke, 2008), or a divine richness in the artwork itself (Hegel, 1993).

Nevertheless, the decision to approach the sublime through experience is not solely based on theoretical considerations. This article illustrates that a profound grasp of the sublime requires one to undergo or witness a certain degree of suffering. Such insight can only be gained through lived experience. Therefore, I argue that engaging with the sublime through experience provides a deeper and more organized comprehension.

In this exploration of the sublime, I aimed to distinguish the formal constituents of the experience of the sublime. In this regard, I have proposed four capital moments of the sublime experience: elevation, mortal atmosphere, images of suffering, and finally, a moment of discovery. These four constituents and the relationships among them, which I have demonstrated, appear to be necessary for such an experience to unfold. Additionally, I connected these four formal elements of meaning to four experiential aspects—upward movement, multiple forms of sensory perception, visual imagery (all accompanied by affectivity), and reflection—that I believe play a key role in the constitution of the experience of the sublime.

Before concluding this article, I would like to make a few remarks about the current state of the art regarding the sublime. It is interesting to note that the classic authors on the sublime—Longinus, Kant, Burke, to name a few—do not seem to agree on their understanding of the term “sublime,” nor do their main theses and ideas about it appear to be part of the same conversation. In this sense, one might suggest that a fitting metaphor to characterize the state of the art on the sublime is that of a labyrinth: when it comes to studying the sublime, following Kant’s path does not guarantee a dialogue with Burke’s main thesis on the matter, and Burke’s thoughts on the sublime do not illuminate Longinus’ ideas on the subject.

That being said, the image of a labyrinth as a metaphor for describing the current state of the discussion about sublimity is much more than just a metaphor. If we reflect on the great literary works that deal with sublimity—Neruda, Woolf, Miller but also the famous works by authors like Dante and Edgar Allan Poe, —the experience of sublimity seems inseparable from navigating a labyrinth; specifically, a labyrinth of sorrow and suffering. Furthermore, one could argue that a key requirement of experiencing sublimity is the ability to endure the difficulties, obscurity, struggle, inconvenience, unexpectedness, awkwardness, chaos, hesitation, and bitterness accompanying suffering. Perhaps it is only through this experience of suffering that developing the new perspective characterizing the idea of the sublime becomes possible.

If the new vision that arises with the experience of the sublime involves enduring a significant amount of suffering, suffering can no longer be regarded as one of the limits of the human condition [as Camus (2005) and Jaspers (1969) believed].^4^ The experience of the sublime seems to emphasize that, within the human condition, there is still something that can be discovered beyond suffering. This “something” that appears to lie beyond suffering can almost immediately be identified in all the great literary accounts of the sublime: sublimity offers a unique perspective on reality. From Dante to Virginia Woolf, from Henry Miller to Poe to Neruda, all the remarkable descriptions of sublimity become possible because reality is now perceived through the lens of wisdom, patience, understanding, and distance gained from experiencing, enduring, and comprehending human suffering, its causes, and its consequences.

“I am coming to speak for and through your dead mouths,” Neruda (2014) wrote. Because when it comes to the sublime, not even death is a limit (but perhaps a starting point). Moreover, Neruda will discuss very unpleasant—terrible—things:

the jeweler with crushed fingers,

the farmer trembling among his seeds,

you, the potter poured in your clay,

all ye, bring to the cup

of this new life

your ancient buried sorrows.

Show me your blood

and your furrow,

tell me: here I was punished

because the jewel did not shine or the earth

failed to yield enough stone

or enough corn:

point to the rock on which you fell

and the wood on which they crucified you

But even more interesting than the aesthetic value of Neruda’s description of horror, suffering, and death is the new perspective— the gaze, the new vision—gained through engaging with suffering, death, and tragedy. “Rise up in birth with me, my brother” is Neruda’s sublime invitation into the history of suffering, torture, violence, and despair that unfolded in Machu Picchu. This is precisely the value of the gaze of the sublime: the universal encounter with the human condition that transcends the limits imposed by time, space, particular emotions, or material existence. Is the experience of the sublime the ultimate boundary of the human condition? It is still hard to determine. Nevertheless, it is difficult not to view the experience of the sublime as one of the ultimate treasures of the human condition, a treasure reserved only for those willing to endure significant suffering and despair.

One might argue that experiencing the sublime is a type of encounter reserved for those who can transcend certain trials that seem to be beyond human tolerance; beyond human condition. In this sense, loss, suffering, tragedy, sorrow, despair, and death appear to be necessary prerequisites for developing the perspective that enables one to approach –and therefore experience—things through the lens of sublimity. Just as Dante’s narrator had to endure hell and purgatory to recognize, appreciate, and ultimately understand the justice, harmony, and perfection of God, all significant accounts of the sublime exhibit a similar structure, particularly the necessity of confronting the limits of human conditions as a prerequisite for experiencing the sublime.

This necessary passage beyond the limits of the human condition, as a prerequisite for experiencing the sublime, is not only represented in classic literary descriptions of the sublime, I would argue. One might even say that all romantic philosophy, to some extent, expresses the sublimation of suffering, death, and sorrow, just as Dostoyevsky’s literature sublimates the experience of injustice, despair, and tragedy.

However, the aftermath of experiencing the sublime is not confined to literature and philosophy. To explore the psychological implications of sublime phenomena, we might inquire: In what way can sublimity contribute to re-signifying tragedy within the realm of psychological treatment? How might the concept of the sublime provide new understandings of how to address suffering and despair? Perhaps a theoretical dialogue between the psychological works of Irvin Yalom and Viktor Frankl and theories of the sublime can open new avenues for psychology and existential therapy.

Finally, I would like to highlight a distinctive feature of the sublime experience. We have noted that this experience involves a confrontation with images of suffering as a prerequisite for being able to experience the sublimity of the encountered place. Experiencing sublimity, in other words, requires preparation to cultivate the perspective that enables us to perceive the sublime.

However, the relationship between atmosphere and the sublime in architectural experience encompasses something deeper. Not only must the subject experiencing architectural sublimity endure images of suffering to realize the sublimity of the architectural construction, but the architectural space itself also grants the subject access to that sublimity. Just as the narrators of Miller or Neruda had to endure the experience of transcending the human condition in order to cultivate the vision that allowed them to grasp the sublime at Manhattan Bridge or in Machu Picchu, these architectural sites inspire them through the sublime atmosphere they evoke. Is there an essential reversibility between the subject’s sublime glance and the sublime atmosphere of the object regarding sublime architecture? Is there a phenomenological correlation between the necessary sublimation of personal experience and the sublime atmosphere of the architectural object within the intentionality of the sublime experience? If so, what are the essential features of this phenomenological correlation? Perhaps the next step for future research on architectural atmosphere is to provide a systematic account of the connections between sublimity in the eye of the beholder and the sublimity of the atmosphere in certain architectural landscapes.

## Data Availability

The original contributions presented in the study are included in the article/supplementary material, further inquiries can be directed to the corresponding author.

## Footnotes

^1^The etymological analysis is not exhaustive as there are many other meanings of the word “elevation” which, for the purpose of keeping the length of the analysis appropriate for the journal, will not be included here. For the same reasons, this analysis is limited to the English language whereas expanding the analysis to include other languages might have given us new insights.

^2^I refer to “space” in a broad sense. As Böhme (2013a) argues, we can speak of the atmosphere not just of a specific physical space, but rather, to atmospheres of various contexts, such as the atmosphere of a dialogue, a zeitgeist, a scenery, a party, an evening, a time period or season.

^3^Interestingly, atmospheres of cities (or urban atmospheres) have been described as largely inter-subjective (Griffero, 2013).

^4^According to Jaspers, suffering constitutes a limit condition due to the inherent confrontation with problems that are ultimately insurmountable. Suffering manifests as an experience wherein individuals discern the existential stakes of their being. The Sublime appears to be among the rare phenomena that transcend the experience of suffering. Thus, following suffering, there exists not merely a void; rather, there emerges the presence of the Sublime.
